# Increased mortality among mothers involved in family court care proceedings compared with their peers in England

**DOI:** 10.23889/ijpds.v9i5.1646

**Published:** 2024-09-18

**Authors:** Georgina Ireland, Linda Wijlaars, Matthew Jay, Ruth Gilbert

**Affiliations:** 1 GOSH Institute of Child Health, University College London

## Objective

Research has called for Family Courts (FC) and social care services involved in removing children from parental care to consider and address the health of the mother.

## Approach

We linked England FC and healthcare data to compare the 10-year mortality risk and cause of death after a first live birth (FLB) for mothers who were involved or not in FC proceedings in England.

## Results

3,072,946 mothers (15-39 years) with a FLB between 2007 and 2019 were followed for up to 10 years (median:8.7 years, IQR: 5.4-10), during which 33,759 were involved in FC. Ten-year cumulative mortality was higher among the FC mothers (1.7 vs 0.3%) and the age-adjusted mortality rate ratio was 7.8 (95%CI: 7.00-8.6).

The majority of maternal deaths in both groups met criteria for avoidable death (FC 82.9% vs no-FC 66.8%). Injury was the leading cause of death (204/420; 48.5%) in mothers involved in FCs and cancer (2,823/6,208; 45.5%) in mothers in the no-FC group. Compared with no-FC mothers, more deaths were related to drug use (35.0% vs 6.0%), suicide (14.5% vs 8.4%) or alcohol (9.8% vs 3.5%).

Among mothers involved in FCs, maternal mortality was higher in cases when children were placed into out-of-home care (1.65%, 463/28143), than when placed at home (0.94%, 53/5563) at first FC proceeding.

## Conclusions and Implications

Addressing high rates of avoidable mortality among mothers involved in FCs is important for children and parents and could be monitored using routinely linked healthcare, social care and family court data.

